# BRCA role changes with association: tissue-specific impact on the prognosis

**DOI:** 10.18632/oncotarget.28149

**Published:** 2021-12-21

**Authors:** Bhavana Gupta, Kumaravel Somasundaram

**Affiliations:** ^1^Department of Microbiology and Cell Biology, Indian Institute of Science, Bangalore-560012, India

**Keywords:** BRCA1, prognosis, tissue-specificity

***Commentary on:** Leaf et al. Opposing effects of BRCA1 mRNA expression on patient survival in breast and colorectal cancer and variation among African American, Asian, and younger patients. Oncotarget. 2021; 12:1992–2005. https://doi.org/10.18632/oncotarget.28082. [PubMed]*

The tumor suppressor BRCA (*BR*east *CA*ncer) genes are majorly involved in biological processes such as DNA damage response, gene transcription regulation, cell cycle, and apoptosis. It was identified in the early 90s that germ line mutations in BRCA1 increase the risk of breast and ovarian cancer [1]. Subsequently, several studies in breast cancer patients found an association of BRCA1 expression levels and mutation status with the patient survival. BRCA genes correlated with favorable overall survival (OS) in some cancers, while opposing effects have also been reported [2]. There are several speculations about this tissue-specificity of BRCA1 gene, but only a few attempts have been made to analyze these theories systematically or experimentally.

The recently published study in Oncotarget [3] contributes to the idea of the tissue-specific impact of BRCA1 expression level on the prognosis of the patient. For this objective, they chose breast cancer (BC) and colorectal cancer (CRC) since there is prior work reporting the conflicting prognostic significance of BRCA1 in these cancers. Authors have stated that “BRCA1 mRNA-low tumor expression positively correlated with BC patient survival but was negatively associated in CRC”. These tissue type-specific associations are explained by the correlation of BRCA1 mRNA expression with different genes in these cancers. In conclusion, the authors show that BRCA1 level correlates with TOP2A and ATAD5 both in BC and CRC and uniquely with LMNB2 in CRC [3].

Our analysis also observed opposing effects of BRCA genes on patient survival outcomes in different cancers. Similar to Leaf et al., 2021, BRCA1 levels correlated with the poor and good prognosis in BC and CRC, respectively [4, 5]. Mei et al., 2020 independently analyzed the prognostic value of BRCA1 using the Kaplan-Meier plotter database in pan-cancer data. In only 5 out of 19 cancers, BRCA1 expression correlated with OS, where two cancers were associated with favorable OS and the remaining three with worse OS supporting the idea of Leaf et al., 2021 [6].

BRCA1 germ line mutations result in altered expression and are reported to affect overall survival. Similar to BRCA1 gene expression, mutants also exhibit tissue-specificity [7]. A study by Zhu et al., 2016 demonstrated that mutation carriers in breast cancer had a poor prognosis compared to Feng et al., 2020 study in CRC, where carriers had favorable responses to chemotherapy [8, 9]. In line with the earlier studies, patients with BRCA1 mutant had poor OS in BC but better OS in CRC.

The idea of tissue-specific opposing effects of BRCA genes on prognosis could be because of their differential interactions with either tumor-suppressor or oncogenes, ultimately affecting the BRCA pathway functional ability. Given the background of BRCA complex behavior and its various biological functions, BRCA1 therapeutic effects may depend on the specificity of the tissue, genetic background, and its multiple mechanisms of action. Further investigation on BRCA interacting spectrum will hopefully elucidate the underlying mechanisms in detail and ultimately help in the advancement of medical interventions.
